# Retarded DNA DSB repair kinetics and augmented radiation sensitivity in Wiskott Aldrich syndrome patients

**DOI:** 10.1038/s41598-026-37262-y

**Published:** 2026-03-16

**Authors:** Ranjana S. Pathak, Rajesh Kumar Chaurasia, Balvinder Kaur Sapra, Pallavi Gaikwad, Umair Bargir, Manisha Madkaikar, Kapil B. Shirsath, Anjana Goel, Nagesh N. Bhat, Arshad Khan

**Affiliations:** 1 Department of Biotechnology, GLA University, Mathura, India; 2 Radiological Physics and Advisory Division, Bhabha Atomic Research Centre (BARC), Mumbai, India; 3 Homi Bhabha National Institute (HBNI), Mumbai, India; 4 Indian Council of Medical Research (ICMR) - National Institute of Immunohaematology (NIIH), KEM Hospital, Mumbai, India

**Keywords:** Wiskott–Aldrich syndrome (WAS), DSB repair kinetics, DSB repair half-time, Genomic instability, Radiosensitivity, Climate sciences, Health care, Medical research

## Abstract

Wiskott–Aldrich Syndrome (WAS), a rare X-linked disorder, features microthrombocytopenia, eczema, immunodeficiency, and elevated malignancy risk due to genomic instability. While prior studies noted DNA repair deficits, the kinetics of ionizing radiation-induced DSB repair in WAS patients remain unclear. This study aimed to characterize DSB repair dynamics and radiation sensitivity in WAS lymphocytes using γH2AX and 53BP1 markers. Lymphocytes from four WAS patients, their carrier mothers, and healthy controls were analyzed. Baseline DSBs were quantified in non-irradiated cells, and repair kinetics assessed post 2 Gy gamma irradiation over 24 h. Immunofluorescence staining for γH2AX (early DSB marker) and 53BP1 (repair facilitator) was performed at multiple time points, with foci quantified via confocal microscopy. Repair half-lives were calculated using exponential decay models. WAS patients exhibited 16–24 fold higher baseline γH2AX and 53BP1 foci than control (mean), indicating spontaneous genomic instability. Post-irradiation, DSB repair in WAS lymphocytes was significantly delayed, with the mean foci repair half-life (T½) in WAS patients being approximately 1.6-fold longer than that of the control (mean). At 24 h post-irradiation, WAS patients retained nearly twice the number of residual foci compared to healthy controls, while carrier mothers mirrored control repair efficiency. This study provides the first evidence of prolonged DSB repair kinetics in WAS patients, emphasising heightened radiosensitivity and genomic instability. These findings suggest tailored radiation strategies in WAS management, particularly for bone marrow transplantation or genotoxic therapies, to mitigate risks and optimize outcomes.

## Introduction

Wiskott–Aldrich syndrome (WAS) was first identified by Alfred Wiskott and Robert Aldrich, who reported that this disease manifests as microthrombocytopenia, eczema and a primary immunodeficiency disorder with various clinical presentations, including susceptibility to autoimmunity and malignancies^1,2^. This X-linked rare disorder primarily affects males, although rare cases of female carriers with variable expressivity have been documented^3^. The incidence is approximately 4 in 1 million live male births in the U.S., with occasional cases occurring in females^4^.

WAS is caused by mutations in the gene encoding the WAS protein (WASp), resulting in both quantitative and qualitative deficiencies in T and B cells. WAS gene is located on the X chromosome (Xp11.22-p11.23)^5^. WASp, which is primarily expressed in hematopoietic cells^6^ regulates actin cytoskeleton dynamics, cell motility, and immune synapse formation,^7,8^. The expression of WASp depends on type and location of mutation in WAS gene^9^. More than 440 genetic mutations have been linked to WAS, with missense mutations being the most common, followed by splice mutations, deletions, and nonsense mutations^10^. Children with WAS have significant risk of developing malignancies, with reported incidences of up to 13%, most commonly EBV-related B-cell lymphomas or leukemia, which typically appear at approximate age of 9.5 y^11^.

DNA double-strand breaks (DSBs), induced by ionizing radiation (IR) or chemotherapeutic agents, represent the most deleterious form of DNA damage, intimidating genomic stability and cell viability if left unrepaired^12,13^. To counteract such lesions, human cells have evolved two major DSB repair pathways: nonhomologous end-joining (NHEJ) and homologous recombination repair (HRR)^14^. Among these, NHEJ serves as the principal and rapid DSB repair mechanism throughout the cell cycle, accounting for most DSB rejoining events outside of S/G₂ phases and even handling the majority of breaks that arise during these phases^15,16^.

In peripheral blood lymphocytes (PBLs), the quiescent, non-dividing G₀/G₁-phase cells analyzed in the present study—DSB repair following irradiation relies almost exclusively on end-joining mechanisms, as these cells lack sister chromatids required for HRR. Consequently, canonical NHEJ (c-NHEJ) and alternative end-joining (alt-NHEJ) pathways, including microhomology-mediated end joining (MMEJ), constitute the only operative routes for DSB repair in such systems^15,17^.

In the broader context of the DNA damage response (DDR), the phosphorylated histone variant γH2AX (phosphorylation of histone H2AX on serine 139) is one of the earliest and most sensitive indicators of DSB formation. Upon DNA damage, the phosphatidylinositol-3-kinase-like kinases (PIKKs) ATM (ataxia-telangiectasia mutated) and DNA-PKcs (DNA-dependent protein kinase catalytic subunit) are rapidly activated, phosphorylating H2AX across megabase chromatin regions surrounding the DSB site^18,19^. γH2AX serves as a chromatin platform that recruits and anchors DDR mediators, coordinating the assembly of repair complexes. The subsequent dephosphorylation and disappearance of γH2AX foci, regulated by phosphatases such as PP2A, PP4C, and WIP1, correlate closely with DSB repair completion and signal termination^20,21^.

Complementary to γH2AX, the tumour suppressor and DDR mediator 53BP1 (p53-binding protein 1) localizes at DSB sites as discrete nuclear foci. 53BP1 modulates DSB repair pathway choice—promoting NHEJ while antagonizing end resection required for HRR. It interacts with γH2AX-marked chromatin through its tandem BRCT domains and other histone modifications, facilitating its accumulation and retention at break sites^22,23^. Disruption of γH2AX-53BP1 signalling compromises the timely recruitment of 53BP1 and delays repair progression. The combined γH2AX/53BP1 co-localization assay has become a gold-standard approach to quantify IR-induced DSBs, track repair kinetics, and assess residual DNA damage in human cells, including quiescent peripheral blood lymphocytes^24,25^.

Recent research has shown the emerging roles of WASH (Wiskott–Aldrich syndrome protein and SCAR homologue) and its intricate components within the nucleus^26–29^. A study conducted in Drosophila cells revealed the essential role of nuclear F-actin, facilitated by the Arp2/3 complex, in relocating heterochromatin breaks to the nuclear periphery for subsequent repair. Interestingly, the depletion of WASH led to a defect in this relocation process, suggesting its potential involvement in DSB repair^30,31^. Moreover, Wang et al. revealed an interaction between WASH and Ku proteins, indicating a regulatory role for WASH in DSB repair pathways in 3T3 mouse fibroblasts^26^.

The timeframe for repairing DSBs is critical for maintaining genomic integrity^32^. In healthy human cells, the standard DSB repair duration typically spans from 2 to 4 h under optimal conditions^33^. However, in instances of impaired DSB repair systems, this timeframe can be significantly prolonged or altered. Previous studies have reported the presence of unrepaired DSBs and genomic instability in cells with WAS mutations^34^. However, the precise kinetics of DSB repair and the half-life of DSB repair processes remain unclear or elusive. The speed and accuracy of DSB repair are vital determinants of cell survival and the prevention of genomic instability and malignancy^35^. Furthermore, they play a crucial role in assessing the sensitivity of WAS patients to genotoxic agents such as radiation, chemotherapeutics, etc^36^.

WAS patients (30–50%) often require Bone Marrow Transplant (BMT) due to immunodeficiency and thrombocytopenia^37^. Conditioning regimens, such as whole-body irradiation, precede BMT to prepare recipients. In WAS, the type and location of the mutation significantly influence disease severity, which in turn affects the degree of radiation sensitivity. This variability in radiation sensitivity is observed among patients with different mutations, reflecting the diverse impact that these genetic changes have on the condition^38^. Studies on DSB repair efficiency and radiation sensitivity in patients with diverse mutations in the WAS gene are currently limited or unavailable. However, leveraging this information could enhance personalized treatment strategies, potentially including dose adjustments based on hypersensitivity assessments, to improve radiation therapy outcomes in this population.

This study investigated the kinetics of DSB repair (utilizing γH2AX and 53BP1 and their colocalization) in the lymphocytes of four WAS patients with different mutations in the WAS gene. The DSB repair half-lives in lymphocytes were estimated, and the presence of spontaneous unrepaired DSBs was measured. However, despite having a heterozygous WAS mutation, carrier mothers exhibited normal repair kinetics. These insights are crucial for evaluating genomic instability and radiation sensitivity and tailoring personalized BMT strategies for the effective management of WAS patients.

## Materials and methods

We evaluated DNA-DSB repair kinetics, quantified repair half-life, and assessed radiation sensitivity in four patients with WAS, their heterozygous mothers, and compared them with healthy controls.

### Ethical approval and sample collection

Institutional ethics committee (IEC) of ICMR- National Institute of Immunohaematology, Mumbai, India has approved this study. Inform consent was obtained to collect peripheral blood samples from patients and family members in heparin vacutainers (make, BD 367884) and the project details were thoroughly explained to them prior to their involvement. The project was conducted in accordance with the ethical guidelines outlined in the declaration.

Written informed consent was obtained from the patients’ guardians for participation in this study and for the publication of the data, with signatures provided accordingly.

### Preparation and irradiation of peripheral blood mononuclear cells (PBMCs)

Peripheral blood mononuclear cells (PBMCs) were isolated from patients, carrier mothers and healthy controls using heparin vacutainers via density gradient centrifugation (using, Histopaque-1077, make Sigma-Aldrich)^39^. After centrifugation, the milky buffy coat containing PBMCs was collected and washed (twice) with RPMI (RPMI 1640 medium, from Gibco). PBMCs containing pellet was resuspended in fresh complete RPMI media. Lymphocytes were then exposed to 2 Gy of ^60^Co-gamma irradiation using a Blood Irradiator (Dose rate: 0.54 Gy/min)^40^. After irradiation, PBMCs were washed and allowed to recover for 24 h in fresh complete RPMI media, in optimum conditions.

### Kinetics of DSB repair foci, γH2AX and 53BP1

The kinetics of DSB repair foci (γH2AX and 53BP1) and their colocalization were investigated for up to 24 h following irradiation, in lymphocytes obtained from the enrolled subjects (WAS patients, carrier mothers and healthy controls). After irradiation, the PBMCs were incubated under optimal conditions (37 °C, 95% relative humidity, and 5% CO_2_) for up to 24 h. PBMCs were sampled at various time points (0, 0.083, 0.17, 0.25, 0.5, 1, 2, 4, 8, 16, and 24 h) post irradiation to quantify the presence of DSB repair foci.

### Immunofluorescence-staining of γH2AX and 53BP1-DSB-repair proteins

Immunofluorescence staining for γH2AX and 53BP1 was performed as previously described (Horn and Rothkamm, 2011; Schultz et al., 2000), with slight modifications^24,41–43^. After post-irradiation incubation, PBMCs were fixed in 4% paraformaldehyde at 4 °C for 30 min, washed with PBS, and deposited on poly-L-lysine-coated coverslips (Corning, BioCoat, Poly-L-Lysine, Glass Coverslip, 354085) to adhere for 1 h. The cells were permeabilized with 0.5% Triton X-100 (make, Sigma-Aldrich), washed, and blocked with 5% FCS (make: Gibco, 10082147) in PBS for 1 h. Primary antibodies against γH2AX (monoclonal, anti-phospho-Histone H2A.X (Ser139) antibody, clone JBW301, mouse-derived; Millipore) and 53BP1 (monoclonal, anti-53BP1 antibody, clone E7N5D, rabbit-derived, Cell Signaling Technology) were added (dilution-1:200), followed by incubation with secondary antibodies (dilution-1:400) (secondary antibody for γH2AX: F(ab’)2-goat anti-mouse IgG (H+L) cross-adsorbed secondary antibody, Alexa Fluor 488, and secondary antibody for 53BP1: polyclonal, donkey anti-rabbit IgG (H + L) highly cross-adsorbed secondary antibody, Alexa Fluor 594, Invitrogen). Cells were mounted using DAPI with antifade solution (ProLong, Diamond Antifade Mountant with DAPI, P36962, Make Invitrogen) for imaging.

### Detection and quantification of γH2AX and 53BP1 foci using confocal microscopy

The mounted slides were imaged using a Leica SP8 confocal fluorescence microscope with Leica Application Suite-X (LAS-X). γH2AX foci were visualized with a green filter, 53BP1 foci with a red filter, and nuclei with a blue filter. 3D z-stack imaging was used to capture foci across all planes, and only spherical nuclei, representing lymphocytes, were included in the analysis. Images were captured and overlaid to show colocalization of γH2AX and 53BP1 in the nuclei. Approximately 300 lymphocytes per individual were examined at each of the 11 incubation time points.

### Statistical analysis

The experiments were performed in three sets, with data presented as mean ± SD. Statistical analysis used Student’s t-test to assess differences among incubation time points and between subjects, with a significance threshold of p < 0.05^44^. Trends were modelled using the least squares method.

## Results

### Case history and pathophysiological conditions of the subjects

In our study, we examined four male paediatric patients (aged 3 months to 14 years), their carrier mothers, and four healthy controls. Clinical, immunological and molecular features of these patients were reported by Gaikwad et al.^9^. These are also summarised in Table 1. Mothers who carried the mutated gene did not manifest symptoms of the disease. Tragically, W1 patient passed away at the age of 5 months due to severe respiratory tract infection and thrombocytopenia. Two patients W2 and W3 are currently suffering from characteristic WAS symptoms and are awaiting BMT. W4 had undergone BMT at the age of 14 years, leading to a notable improvement in his quality of life.

**Table 1:** Depiction of pathophysiological symptoms, age, mutation details, and current status of the four patients diagnosed with WAS.

Subjects	Patients code	Age and gender	Symptoms	Mutation details	Current status
W1	P31	5 months (male)	Born with second degree consanguineous marriage. At 4 months of age c/o cough, fever and increased respiratory activity, thrombocytopenia. Presented with eczema and bloody stools	Frameshift c.1266_1267insG (p.L425Pfs*70)	Expired
W2	P40	3 months (male)	Born with non-consanguineous marriage, presented with history of repeated episodes of loose stool with blood, recurrent sepsis, anaemia with thrombocytopenia. Cytomegalovirus (CMV) positive. Upper GI endoscopy and ileocolonoscopy shows gastritis and pancolitis. Bone marrow aspiration showed normal results	Frameshift c.763_764insG (p. Q255Rfs*5)	Awaiting BMT
W3	P39	1 year (male)	Eczema, thrombocytopenia, Lower Respiratory Tract Infections (LRTI) with primary compared to their respective baselinegen requirement	Missense c.134C > T (p. T45M)	Awaiting BMT
W4	P21	14 years (male)	H/o bleeding manifestation (skin and mucosal bleeding) and recurrent respiratory tract infection and H/o thrombocytopenia	Missense c.223G > C (p.V75L)	Transplanted
Mother of W1	MW1	Age not recorded	Asymptomatic, no clinical manifestations	Mutation analysis not performed	Clinically normal
Mother of W2	MW2	Age not recorded	Asymptomatic, no clinical manifestations	Mutation analysis not performed	Clinically normal
Mother of W3	MW3	Age not recorded	Asymptomatic, no clinical manifestations	Mutation analysis not performed	Clinically normal
Mother of W4	MW4	Age not recorded	Asymptomatic, no clinical manifestations	Mutation analysis not performed	Clinically normal
Control 1	C1	22 years (male)	Healthy control with no reported clinical symptoms	Mutation analysis not performed	Clinically normal
Control 2	C2	28 years (male)	Healthy control with no reported clinical symptoms	Mutation analysis not performed	Clinically normal
Control 3	C3	31 years (male)	Healthy control with no reported clinical symptoms	Mutation analysis not performed	Clinically normal
Control 4	C4	40 years (male)	Healthy control with no reported clinical symptoms	Mutation analysis not performed	Clinically normal

For comparison, the age, health conditions, and symptoms of their carrier mothers and four healthy controls are also presented*.*

### Baseline DSB (genomic instability) assessment

**Fig. 1:**
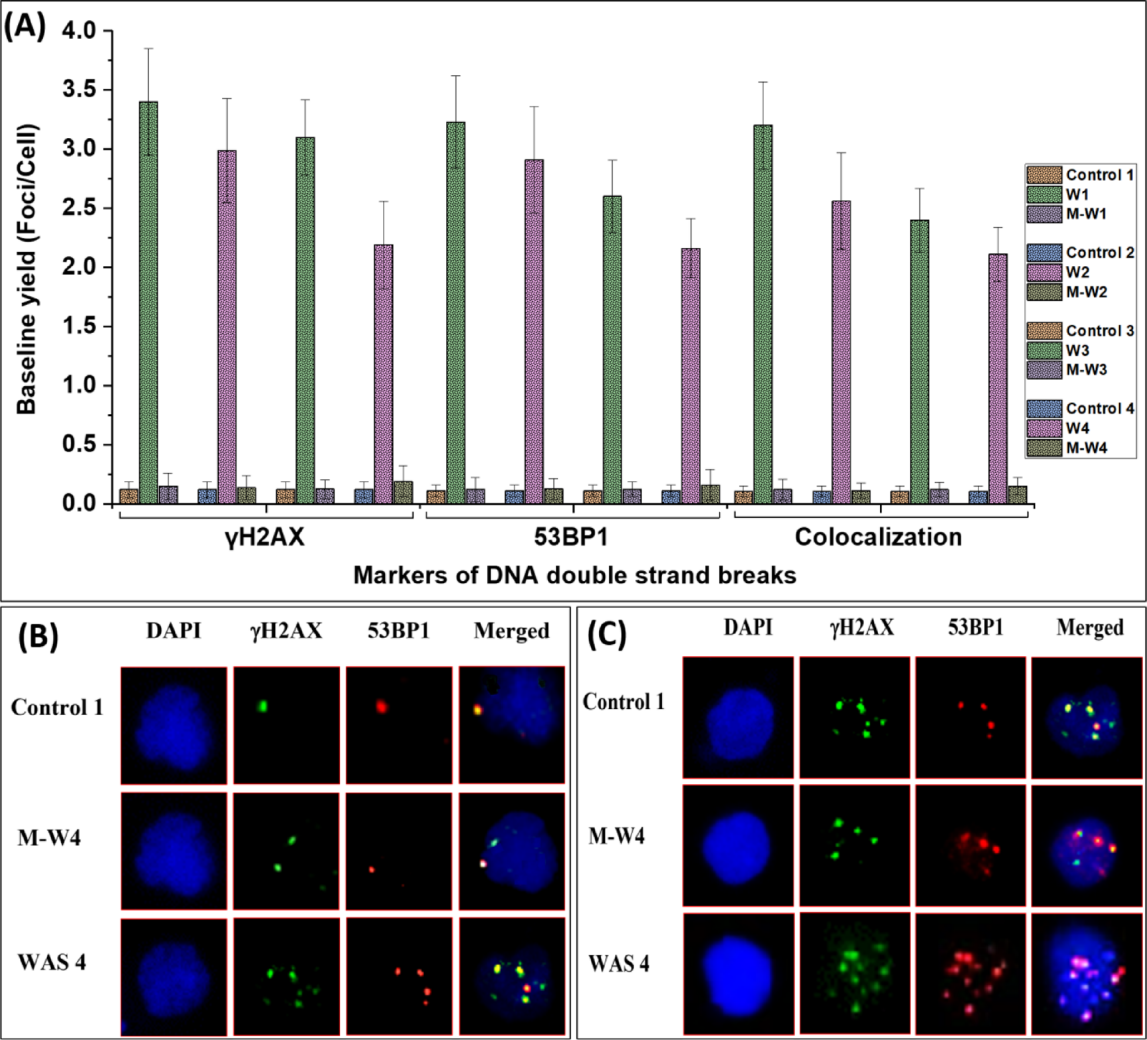
(**A**) Bar graph depicting baseline frequencies of γH2AX and 53BP1 foci, and their colocalization, in lymphocytes from four WAS patients, their carrier mothers, and four healthy controls. (**B**) Representative confocal images showing baseline γH2AX (green) and 53BP1 (red) foci, and their colocalization (yellow), in lymphocytes from a healthy control (control 1), the carrier mother of WAS patient 4 (MW4), and WAS patient 4 (W4). WAS patient exhibited significantly higher foci frequencies than control, while carrier mother’s frequencies aligned with control. (**C**) Representative images of lymphocytes 24 h post-irradiation, illustrating residual γH2AX and 53BP1 foci, and their colocalization. WAS-4 patient lymphocytes displayed markedly more residual foci than carrier mother (MW4) and control (control 1), indicating persistent unrepaired DSBs.

Results showed significantly elevated frequencies of γH2AX and 53BP1 foci in the lymphocytes of WAS patients (γH2AX: 16–24 times and 53BP1: 17–25 times) compared to those in the healthy control (mean) (Table 2; Fig. 1A). The frequencies of foci observed in the mothers were found to be within the range of the controls. Figure 1B is a representative image depicting the foci and their colocalization in the lymphocytes of a healthy control volunteer (control 1), the WAS patient (WAS 4), and a carrier mother (mother of WAS 4). A good colocalization (71–99%) between γH2AX and 53BP1 foci was observed among the subjects. The observed background levels were in agreement with previous reports^45,46^, with spontaneous foci in healthy control lymphocytes measured as γH2AX: 0.10 ± 0.03 and 53BP1: 0.12 ± 0.02 foci per nucleus.

**Table 2:** Baseline data on γH2AX and 53BP1 foci/cell and their colocalization in lymphocytes from four WAS patients, their carrier mothers and four healthy controls.

Subjects	Baseline levels of foci/cell		
	γH2AX foci/cell	53BP1 foci/cell	Colocalization foci/cell
WAS1 (W1)	3.4 ± 0.45	3.23 ± 0.39	3.2 ± 0.37
WAS2 (W2)	3.1 ± 0.32	2.6 ± 0.31	2.4 ± 0.27
WAS3 (W3)	2.19 ± 0.37	2.16 ± 0.25	2.11 ± 0.23
WAS4 (W4)	2.99 ± 0.44	2.91 ± 0.45	2.56 ± 0.41
Mean of W1–W4	2.92 ± 0.52	2.73 ± 0.46	2.57 ± 0.46
Mother of W1	0.147 ± 0.112	0.121 ± 0.101	0.118 ± 0.087
Mother of W2	0.135 ± 0.102	0.123 ± 0.089	0.112 ± 0.065
Mother of W3	0.123 ± 0.081	0.121 ± 0.063	0.12 ± 0.062
Mother of W4	0.189 ± 0.131	0.159 ± 0.131	0.149 ± 0.072
Mean of MW1–MW4	0.149 ± 0.029	0.131 ± 0.019	0.125 ± 0.017
Control 1 (C1)	0.141 ± 0.037	0.134 ± 0.041	0.121 ± 0.037
Control 2 (C2)	0.139 ± 0.068	0.129 ± 0.049	0.119 ± 0.045
Control 3 (C3)	0.137 ± 0.029	0.131 ± 0.031	0.123 ± 0.032
Control 4 (C4)	0.138 ± 0.053	0.126 ± 0.043	0.114 ± 0.051
Mean of C1–C4	0.139 ± 0.002	0.13 ± 0.003	0.119 ± 0.004

### DSB repair dynamics and radiation sensitivity in lymphocytes of WAS patients

**Fig. 2:**
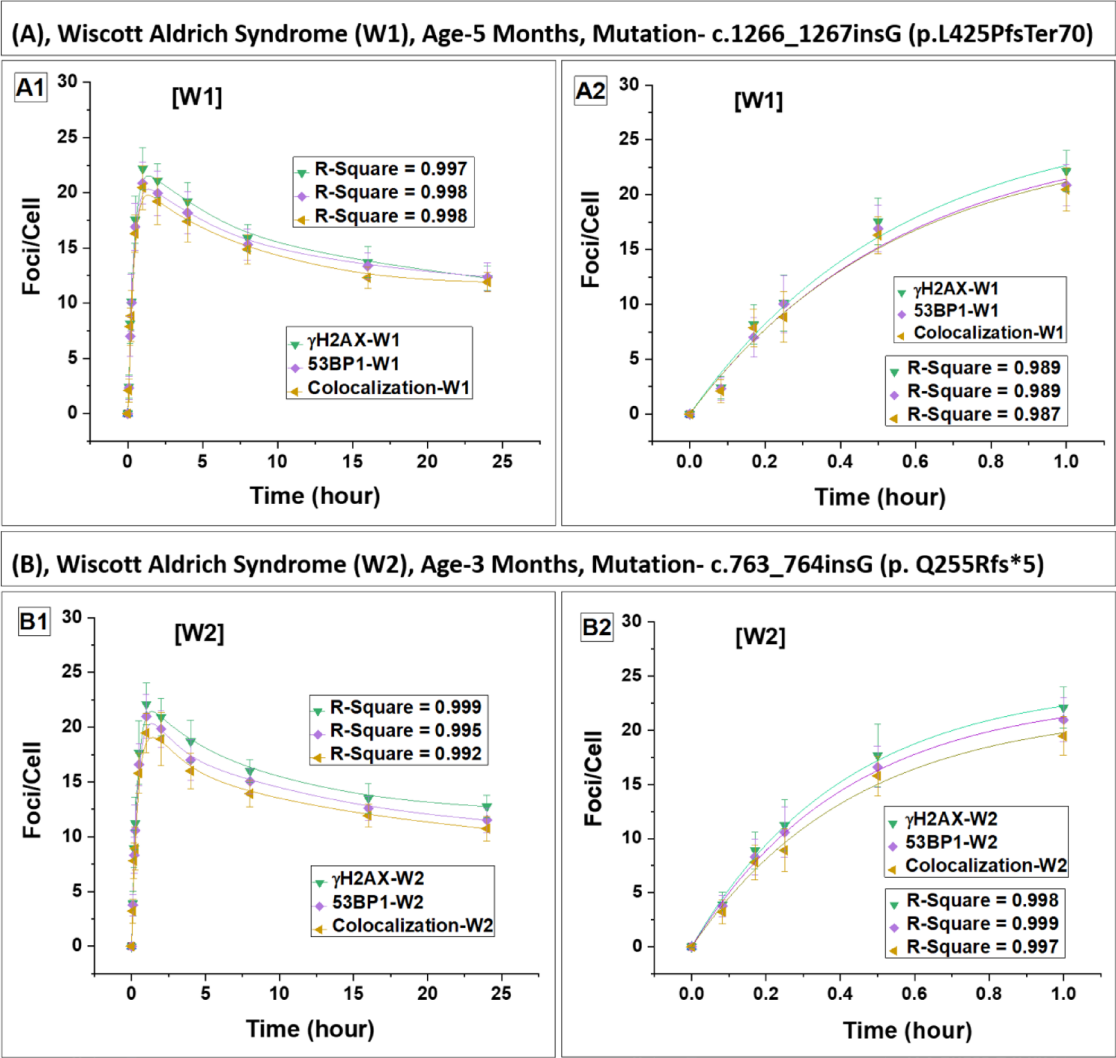

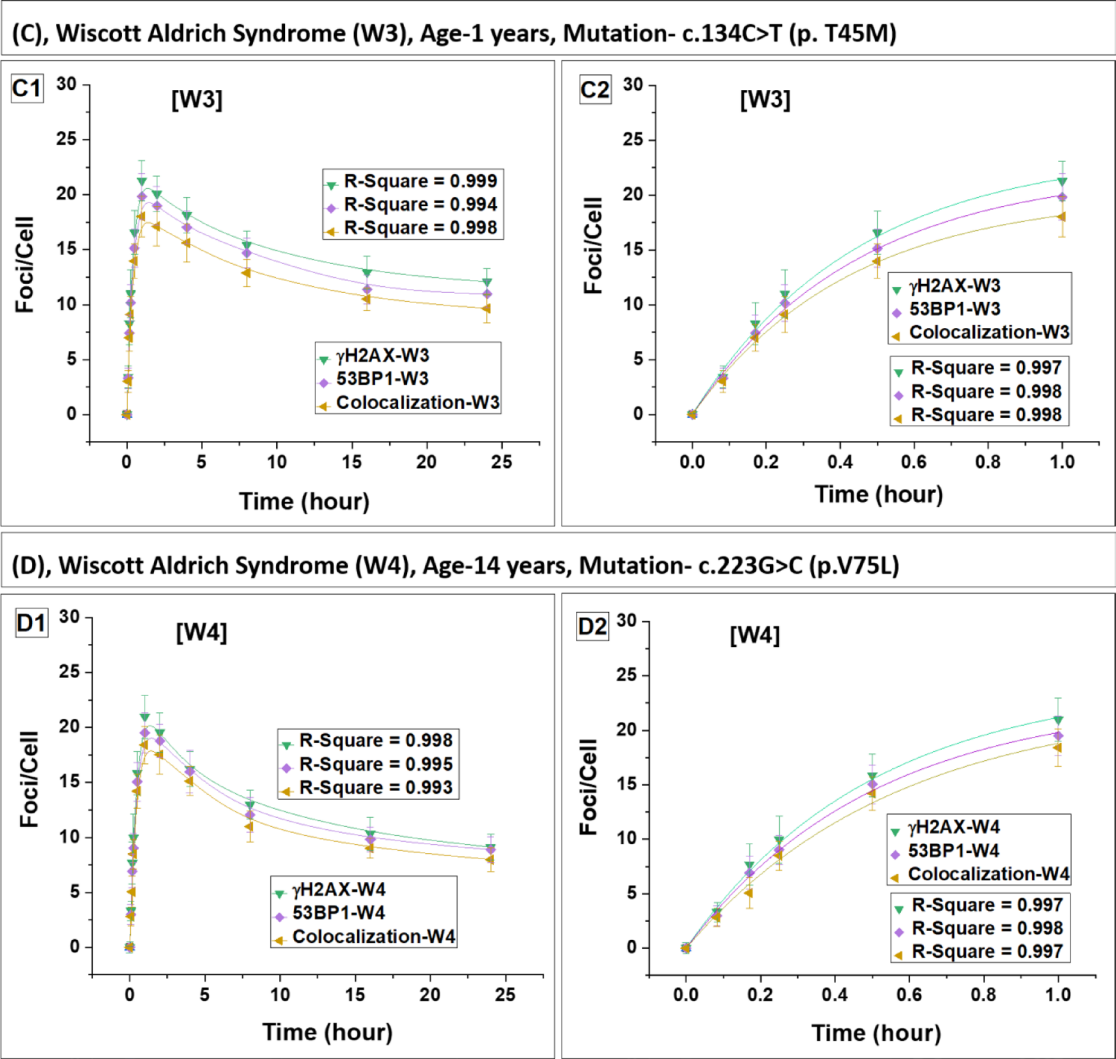
Kinetics of foci (γH2AX, 53BP1 and their colocalization) buildup and decay in four WAS patients. Panels (**A**–**D**) represent data for patients W1, W2, W3 and W4, respectively. Foci quantification was carried out from 0 to 24 h postirradiation. Error bars represent the standard deviation (SD) with a sample size of n = 3 × 3 and the number of cells scored per sample was N = 100. The data presented here have been baseline-corrected for foci.

**Fig. 3:**
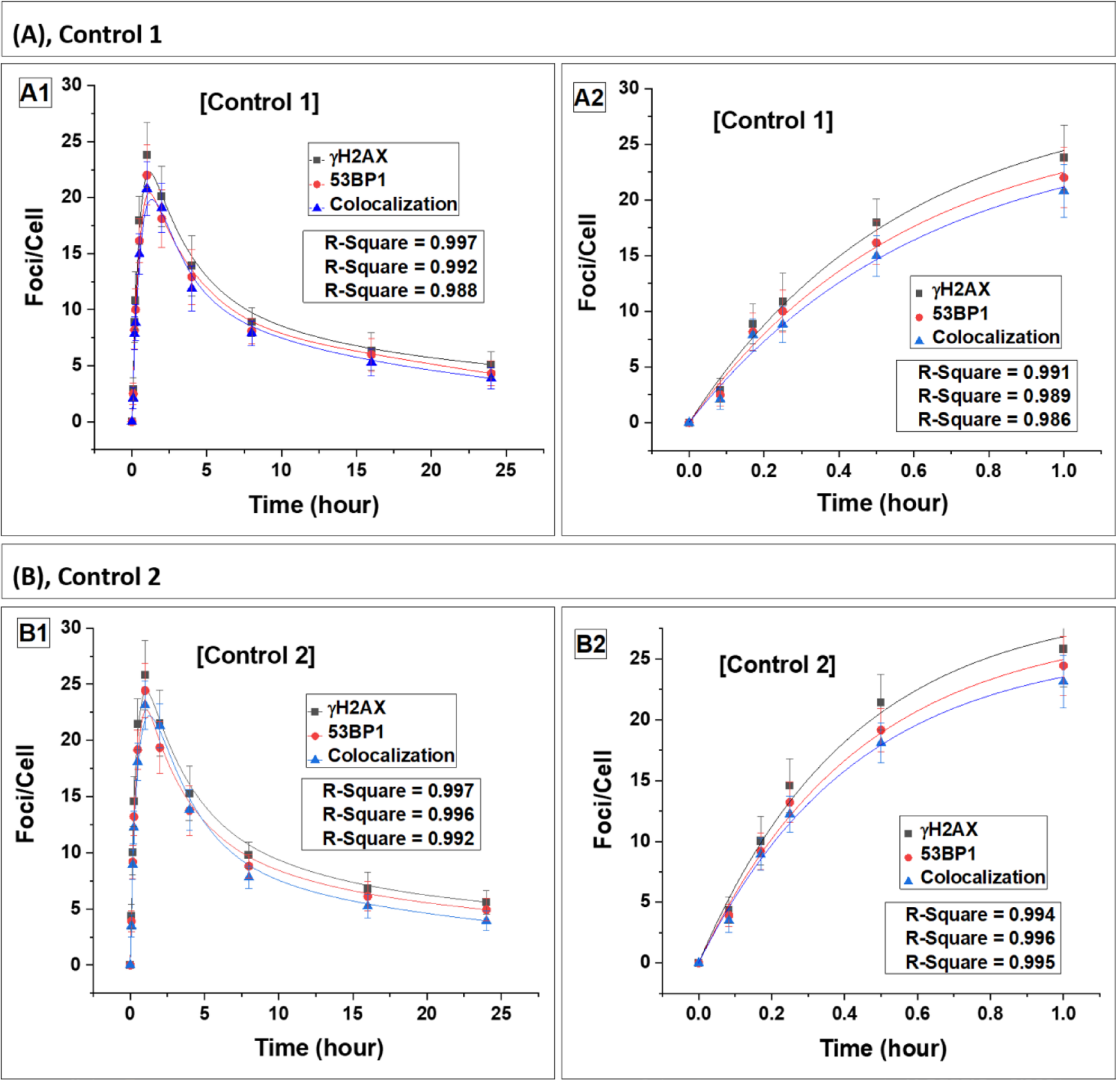

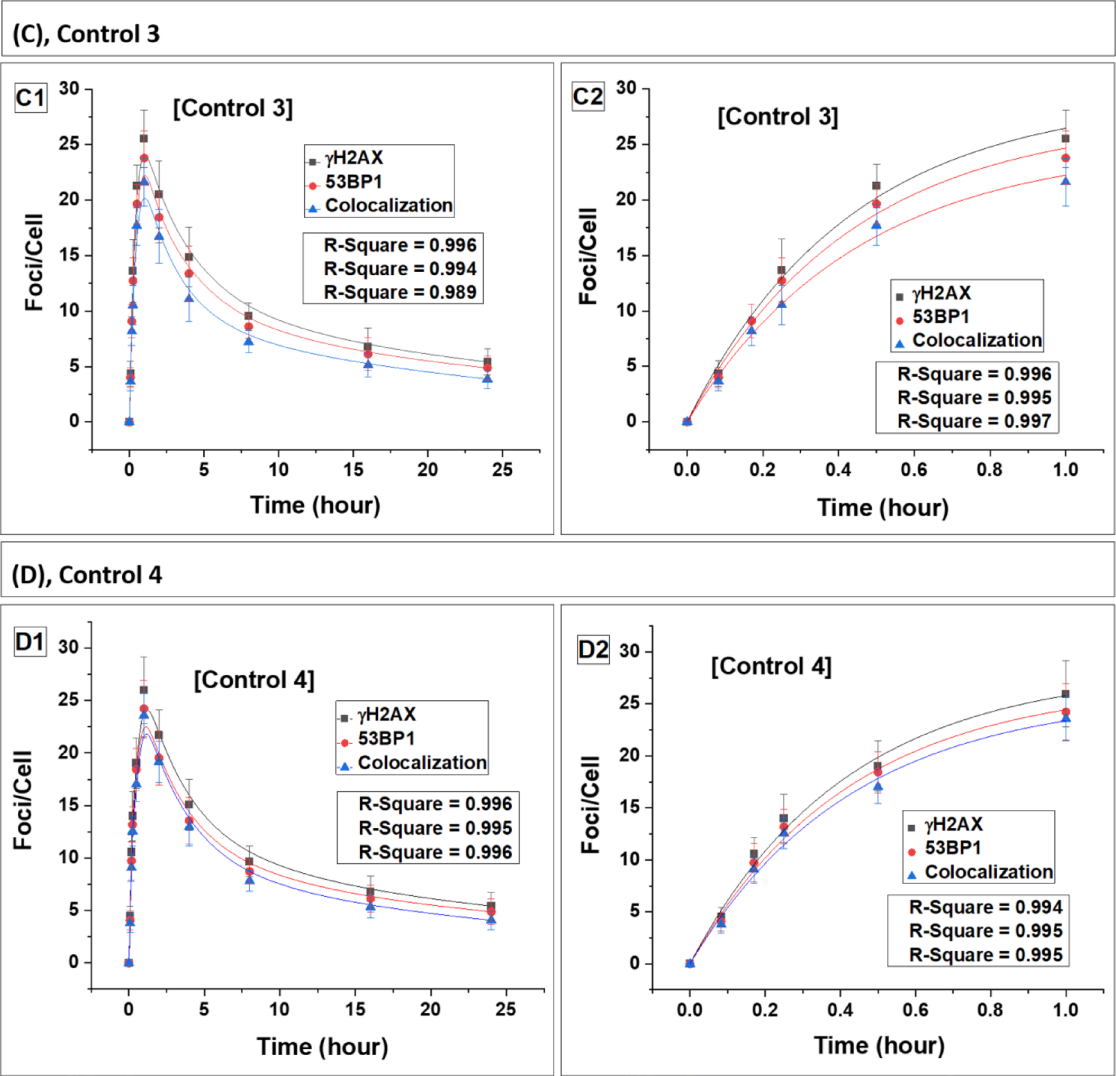
Kinetics of foci (γH2AX, 53BP1 and their colocalization) buildup and decay in the healthy controls (C1–C4). Panels (**A**–**D**) represent data for four healthy controls, C1, C2, C3 and C4, respectively. Foci quantification was carried out from 0 to 24 h post irradiation. Error bars represent the standard deviation (SD) with a sample size of n = 3 × 3 and the number of cells scored per sample was N = 100. The data presented here have been baseline-corrected for foci.

We have assessed abundance of γH2AX and 53BP1 foci, as well as their colocalization post gamma exposure in WAS patients (Fig. 2) along with four healthy controls (Fig. 3). Within 15 min post-irradiation, foci were detectable, with a statistically significant increase (p < 0.05) compared to their respective baseline levels in all subjects. The fitted curves are represented in Fig. 2A–D. While Fig. 1C illustrates foci and their colocalization in the lymphocytes of a healthy control volunteer (control 1 is shown in the figure), the WAS4 patient, and carrier mother (mother of WAS 4 is shown in the figure), 24 h post-irradiation.

**Fig. 4:**
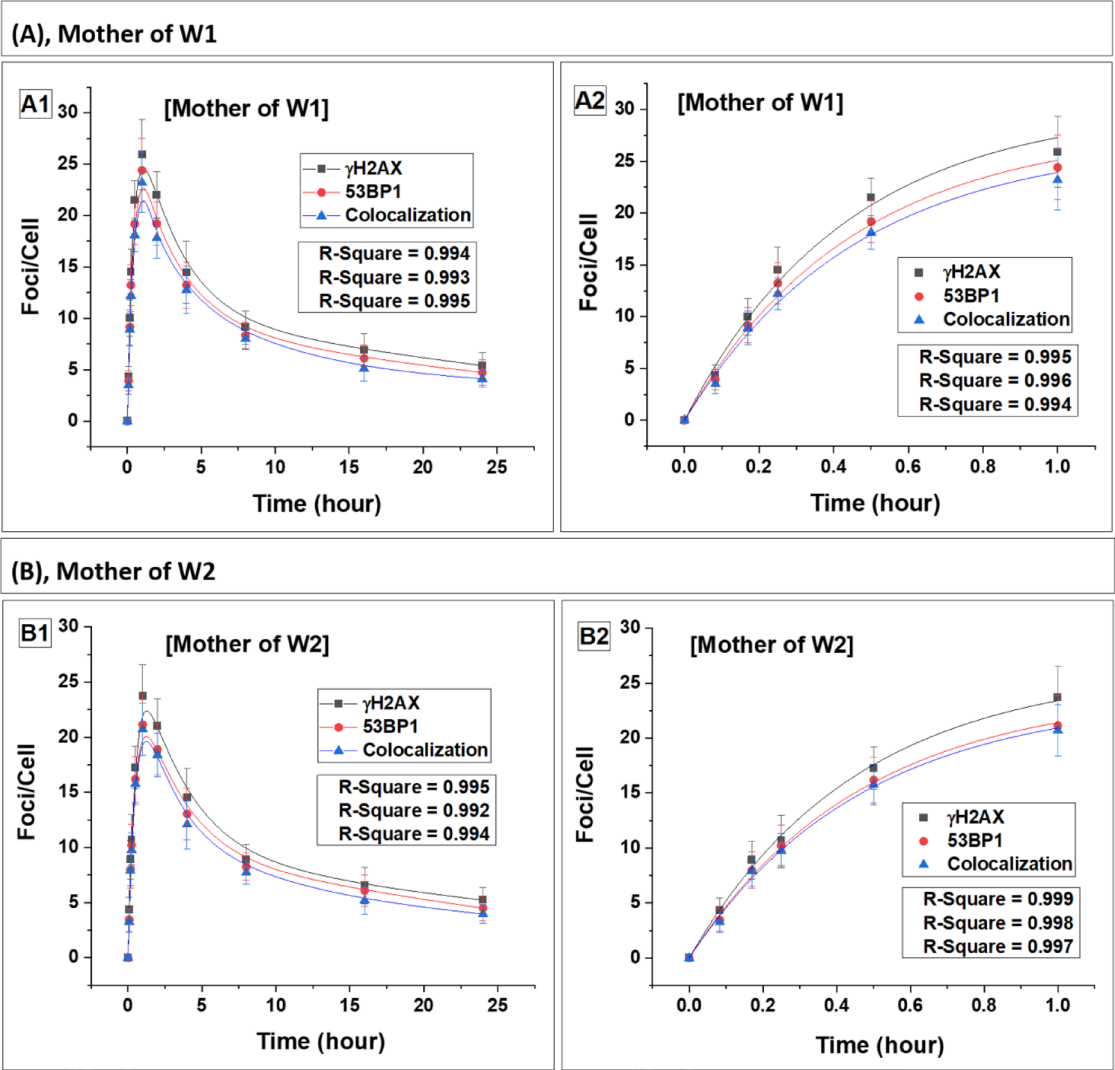

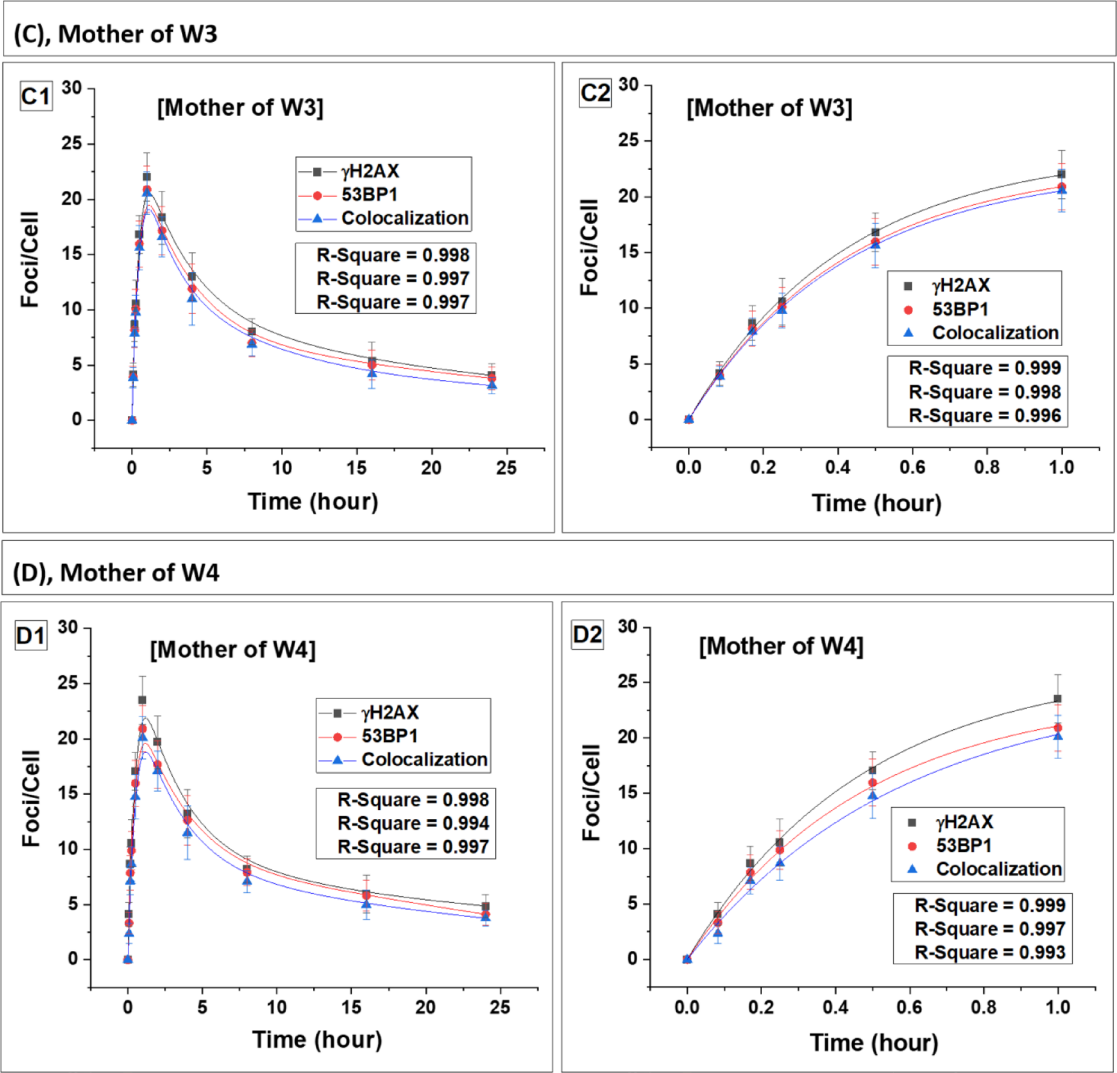
Kinetics of foci (γH2AX, 53BP1 and their colocalization) buildup and decay in the carrier mother (MW1-MW4). Panels (**A**–**D**) represent data for carrier mothers, MW1, MW2, MW3 and MW4, respectively. Foci quantification was carried out from 0 to 24 h post irradiation. Error bars represent the standard deviation (SD) with a sample size of n = 3 × 3 and the number of cells scored per sample was N = 100. The data presented here have been baseline-corrected for foci.

The fitting analysis revealed a pattern where the number of foci initially increased and then followed a saturating exponential function, which can be expressed as $\mathrm{Y}=\mathrm{A}(1-{\mathrm{e}}^{-\mathrm{kt}})$ where A, and k are constants. Similar pattern was reported earlier in control samples^24,41^. The units of Y and A, are foci/cell, while k is expressed in h^−1^ and t represents time in h. The constant k, representing the rate of foci formation, showed significant variation among the four WAS patients and they exhibited a lower rate of foci formation compared to control (mean) (Table 3) and their carrier mother (mean). Foci formation reached saturation after 1 h in all subjects with a plateau observed up to 2 h post-irradiation. Subsequently, the yield of foci began to decrease in both WAS and control lymphocytes. The average saturation yield of the foci was slightly lower in WAS patients compared to control (mean) and carrier mother (mean).

**Table 3:** Fitting parameters for the build-up of foci (γH2AX, 53BP1, and their colocalization) in lymphocytes obtained from four WAS patients and their carrier mothers, along with four healthy controls.

Subjects	Build-up constant (k) of γH2AX, 53BP1, and colocalized foci in lymphocytes of WAS patients, carrier mothers, and healthy controls		
	γH2AX foci	53BP1 foci	Colocalization of foci
	Build up constant k (h^−1^)	Build up constant k (h^−1^)	Build up constant k (h^−1^)
W1	1.79 ± 0.47	1.78 ± 0.47	1.83 ± 0.54
W2	2.45 ± 0.21	2.40 ± 0.19	2.29 ± 0.29
W3	2.26 ± 0.26	2.26 ± 0.21	2.37 ± 0.21
W4	2.01 ± 0.19	1.99 ± 0.20	1.78 ± 0.26
Mean of W1–W4	2.12 ± 0.29	2.11 ± 0.28	2.07 ± 0.31
MW1	2.32 ± 0.39	2.23 ± 0.35	2.19 ± 0.39
MW2	2.21 ± 0.17	2.16 ± 0.22	2.14 ± 0.25
MW3	2.39 ± 0.15	2.37 ± 0.13	2.35 ± 0.12
MW4	2.11 ± 0.15	2.11 ± 0.22	1.74 ± 0.34
Mean of MW1–MW4	2.26 ± 0.12	2.22 ± 0.11	2.11 ± 0.26
C1	1.76 ± 0.43	1.72 ± 0.45	1.62 ± 0.51
C2	2.38 ± 0.39	2.28 ± 0.34	2.33 ± 0.35
C3	2.34 ± 0.41	2.30 ± 0.32	2.23 ± 0.26
C4	2.42 ± 0.37	2.37 ± 0.36	2.33 ± 0.36
Mean of C1–C4	2.23 ± 0.31	2.17 ± 0.30	2.13 ± 0.34

The lymphocytes were exposed to 2 Gy of ^60^Co-γ-rays, and foci were quantified up to 1-h post-irradiation. Initial foci build up followed a single exponential growth pattern, mathematically expressed as $\mathrm{Y}=\mathrm{A}(1-{\mathrm{e}}^{-\mathrm{kt}})$.

A comparative analysis of the mean foci build-up constant (k) showed that WAS patients exhibited a lower rate of foci formation compared to both carrier mothers and healthy controls (Table 3). The mean k-value for WAS subjects was slightly lower relative to carrier mothers and controls. Statistical comparison (unpaired t-test) confirmed that the difference between WAS vs carrier mothers and WAS vs controls was significant (p < 0.05), whereas no significant difference was observed between carrier mothers and controls (p > 0.05), suggesting a normal foci kinetics in carrier mothers.

In a similar manner, the kinetics of foci decay were examined from 2 to 24 h of irradiation, and the data were best fitted with a single exponential decay pattern given by: $\mathrm{Y}={\mathrm{Y}}_{0}+{\mathrm{A}}_{1}{\mathrm{e}}^{-\mathrm{k}1\mathrm{t}}$. This decay model does not differentiate between fast and slow decay components individually; it combines both into a single component defined by the constant k1, consistent with earlier findings^24,41^. Parameter Y_0_, A_1_, k1 and t, represent residual foci, peak foci, foci decay constant and time in h.

Comparative analysis of the decay constants (k₁) demonstrated that the rate of foci decay in WAS patients was approximately 1.5–1.9 times slower than in healthy controls (Table 4), as confirmed by Student’s t-test (p < 0.01), indicating a significant delay in DSB repair kinetics. A slight variation is observed in the decay rates of foci among WAS patients indicative of the dependence of the repair on the type of gene mutation. Furthermore, at 24 h post-irradiation, WAS patients retained nearly twice the number of residual foci compared to healthy controls (p < 0.01, Student’s t-test), suggesting persistence of unrepaired or slowly repairing DSBs. These findings clearly indicate a compromised DNA-DSB repair capacity in WAS lymphocytes relative to healthy controls.

**Table 4:** Depiction of the decay constant (k₁) and DSB repair half-life (T₁_/_₂) for foci (γH2AX, 53BP1, and colocalized foci) in lymphocytes from four WAS patients, their carrier mothers, and four healthy controls.

Subjects	Decay constants (K1) of γH2AX, 53BP1, and their colocalized foci, and estimation of DNA-DSB repair half-life (T₁/₂) in lymphocytes of four WAS patients, their carrier mothers, and four healthy controls					
	γH2AX foci		53BP1 foci		Colocalization of foci	
	Decay constant k1 (h^−1^)	DSB repair half-life T_1/2_	Decay constant k1 (h^−1^)	DSB repair half-life T_1/2_	Decay constant k1 (h^−1^)	DSB repair half-life T_1/2_
W1	0.126 ± 0.012	5.50 ± 0.59	0.136 ± 0.011	5.11 ± 0.46	0.146 ± 0.011	4.73 ± 0.39
W2	0.142 ± 0.002	4.89 ± 0.07	0.134 ± 0.015	5.16 ± 0.67	0.138 ± 0.019	5.04 ± 0.79
W3	0.134 ± 0.001	5.18 ± 0.05	0.118 ± 0.016	5.88 ± 0.92	0.12 ± 0.007	5.77 ± 0.37
W4	0.156 ± 0.011	4.43 ± 0.33	0.155 ± 0.019	4.48 ± 0.62	0.15 ± 0.019	4.62 ± 0.71
Mean of W1–W4	0.139 ± 0.013	5.00 ± 0.45	0.136 ± 0.015	5.16 ± 0.57	0.138 ± 0.013	5.04 ± 0.52
MW1	0.255 ± 0.029	2.72 ± 0.31	0.259 ± 0.029	2.68 ± 0.30	0.225 ± 0.021	3.08 ± 0.28
MW2	0.232 ± 0.021	2.99 ± 0.27	0.216 ± 0.027	3.21 ± 0.39	0.217 ± 0.022	3.19 ± 0.31
MW3	0.218 ± 0.014	3.18 ± 0.20	0.243 ± 0.019	2.85 ± 0.22	0.224 ± 0.017	3.09 ± 0.23
MW4	0.254 ± 0.016	2.73 ± 0.18	0.215 ± 0.022	3.23 ± 0.36	0.228 ± 0.017	3.04 ± 0.25
Mean of MW1–MW4	0.239 ± 0.018	2.91 ± 0.22	0.233 ± 0.022	2.99 ± 0.27	0.224 ± 0.005	3.10 ± 0.06
C1	0.232 ± 0.015	2.98 ± 0.2	0.225 ± 0.022	3.08 ± 0.34	0.216 ± 0.028	3.21 ± 0.47
C2	0.227 ± 0.013	3.05 ± 0.18	0.237 ± 0.020	2.92 ± 0.25	0.230 ± 0.027	3.01 ± 0.35
C3	0.229 ± 0.018	3.03 ± 0.24	0.239 ± 0.024	2.89 ± 0.29	0.258 ± 0.034	2.69 ± 0.35
C4	0.233 ± 0.019	2.97 ± 0.25	0.243 ± 0.022	2.85 ± 0.25	0.244 ± 0.020	2.84 ± 0.24
Mean of C1–C4	0.230 ± 0.003	3.01 ± 0.04	0.236 ± 0.008	2.94 ± 0.10	0.237 ± 0.018	2.94 ± 0.22

Lymphocytes were exposed to 2 Gy of ^60^Co-γ-rays, and foci were quantified up to 24 h post-irradiation. Foci decay kinetics followed a single exponential decay pattern, expressed as $\mathrm{Y}={\mathrm{Y}}_{0}+{\mathrm{A}}_{1}{\mathrm{e}}^{-\mathrm{k}1\mathrm{t}}$. The half-lives (T_1/2_) of DSB repair foci (γH2AX, 53BP1, and their colocalization) were estimated for the aforementioned subjects using first-order kinetics, with T_1/2_ = 0.693/k1.

The half-lives (T_1/2_) of DSB repair foci in both WAS and control lymphocytes were determined using first-order kinetics, calculated with the expression T_1__/2_ = 0.693/k1^24,41^. Among four WAS patients, a slight variation in the half-lives of the foci and their colocalization was observed.

The mean foci repair half-life (T½) in WAS patients was approximately 1.6-fold longer compared to the mean of the controls (Table 4). Statistical analysis using Student’s t-test confirmed that this delay in DSB repair was significant (p < 0.01). This difference in repair efficiency of WAS patients raises significant concerns regarding genomic stability in WAS patients, potentially heightening their vulnerability to radiation exposure and increasing their risk of developing malignancies.

### DSB repair dynamics and radiation sensitivity in carrier mothers of WAS patients

The dynamics of DSB repair foci were also examined in the lymphocytes of carrier mothers (MW1-MW4) (Fig. 4). Data for the carrier mothers of all four WAS patients are presented in Table 3 (foci buildup kinetics) and Table 4 (foci decay kinetics). Statistical comparison using Student’s t-test showed no significant difference between carrier mothers and controls in both induction and decay parameters (p > 0.05), indicating comparable response patterns.

The foci induction and decay kinetics in carrier mothers closely paralleled those observed in the controls, with approximately 20% residual foci persisting at 24 h, consistent with the control mean values (Table 3) (p > 0.05, Student’s t-test). The estimated repair half-lives (T½) of the foci in lymphocytes of carrier mothers were also statistically indistinguishable from those of the control group (Table 4) (p > 0.05). These findings collectively indicate that carrier mothers of WAS patients retained wild-type DSB repair efficiency and did not exhibit features of increased radiation sensitivity.

## Discussion

**Fig. 5:**
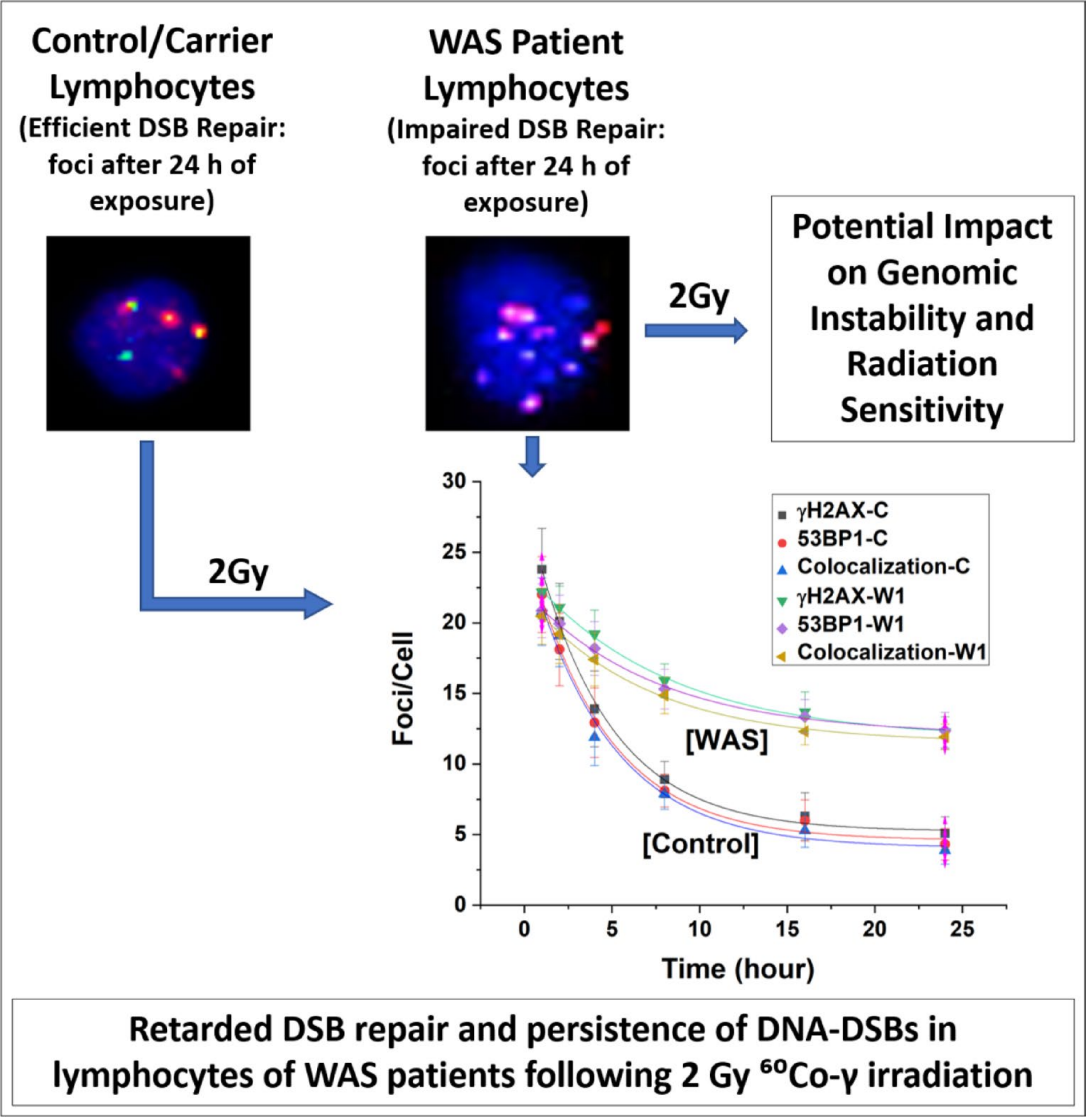
Graphical representation of impaired DNA-DSB repair in lymphocytes from WAS patients, compared to lymphocytes from a healthy control volunteer. The illustration highlights the reduced DSB repair efficiency in WAS lymphocytes relative to controls.

Previous reports have indicated that the pathophysiological conditions and severity of WAS patients vary significantly depending on the type and location of the mutation in the WAS gene^38^. This study investigated the effects of radiation exposure on genome instability in four WAS patients possessing different WAS gene mutations. For the first time, this study evaluates DNA-DSB repair kinetics in WAS patients and their carrier mothers, employing γH2AX and 53BP1 foci assay. We have noted ~ 16 to ~ 24 times elevated baseline levels of DSB markers (γH2AX and 53BP1) in lymphocytes of WAS patients compared to their carrier mothers and four healthy controls (graphically summarised in Fig. 5). A similar result has been reported by Wang et al. in in vitro WAS knockout cells^26,47,48^. The baseline occurrence of DSBs arises from various endogenous and exogenous factors, triggering the generation of reactive oxygen species (ROS) and consequent DSBs in cells^49,50^. Cellular processes, such as cell division and cell differentiation, can also provoke the formation of DSBs^51^. Wild-type cells have the ability to repair these spontaneously induced DSBs, preventing their accumulation. However, WAS-mutant cells exhibit impaired DSB repair, potentially leading to the accumulation of these DSBs over time^26,36^.

Typically, WAS-mothers demonstrate normal basal levels of expression of DSB repair foci, although in rare instances, higher spontaneous yields have also been reported^3^. However, our results demonstrated that the repair yield (at 24 h) of DSBs, indicated by γH2AX and 53BP1 foci, in the mothers was consistent with the typical range observed in controls and other documented human populations (0 to 0.49 foci/cell in populations from Germany, France, Cuba, India, and elsewhere)^42,43,52,53^.

Recent studies have elucidated the critical role of WAS protein (WASP) in DNA repair mechanisms, particularly emphasizing DSB repair^6,10,11,26,36^. WASP deficiency hampers the assembly of actin filaments at DNA damage sites, impeding the recruitment of repair factors and compromising the competence of DSB repair pathways, including HRR, NHEJ, and other end-joining pathways (MMEJ, Alt-NHEJ); however, HRR is largely non-functional in PBLs because these cells are in the quiescent phase of the cell cycle.^36,54,55^. Moreover, a recent study demonstrated that WASp directly interacts with RPA, enhancing its binding to single-stranded DNA and promoting efficient DNA repair and replication fork stability, whereas WASp deficiency impairs RPA function, leading to defective repair and increased genomic instability^56^.

The kinetics of DSB repairs, such as the repair half-life and the dynamics of repair foci over time, are not adequately understood or explored in WAS lymphocytes. To assess the radiation sensitivity and genomic stability, DSBs were created by irradiating WAS lymphocytes with 2 Gy of gamma radiation and foci (γH2AX and 53BP1) formation and decay kinetics was studied. This study demonstrated that WAS lymphocytes exhibit a similar trend in the DSB repair process as control lymphocytes, with an initial rapid repair phase followed by a slower phase. Though, the pace of DSB repair was significantly reduced in WAS-lymphocytes than that in the controls. Results showed presence of significantly higher residual foci after 24 h reflecting the persistence of excess DSBs in WAS lymphocytes, compared to healthy control cells. These findings clearly indicate the accumulation of unrepaired DSBs in radiation-challenged WAS lymphocytes, suggesting increased radiosensitivity and genomic instability.

Different levels of radiosensitivity were observed among the four WAS patients (Table 4). W1 exhibited the highest level of radiosensitivity, with a ~ 1.8-fold greater DSB repair half-life (slowest pace of DSB repair) than that of the control (mean), as evidenced by the data on γH2AX foci. Conversely, patient W4 exhibited a relatively lower level of sensitivity, with a ~ 1.5-fold increase in the half-life of DSB repair compared with that of the control (mean). This differential sensitivity was due to frameshift mutations, as observed in W1, significantly impair WAS function compared to the milder impact of missense mutations, such as those seen in W4.

Repairing complex DSBs is time-intensive, often taking hours to days. In contrast, 2 Gy of low-LET radiation (^60^Co-γ-ray) majorly induces simpler DSBs, usually repaired within 2–4 h^57^. This study showed that WAS patients exhibit incomplete repair of simple DSBs for up to 24 h (Table 4) of DSB induction.

Radiation exposure can result into compromised immune function, increasing susceptibility to infections and other complications^36^. Understanding the extent of radiation sensitivity in WAS patients can guide healthcare providers in optimizing infection prevention strategies and providing timely interventions to minimize adverse effects.

Owing to the accumulation of DSBs and genomic instability, WAS patients face an increased risk of developing malignancies^48^. Radiation therapy is a commonly used treatment modality for various malignancies and autoimmune conditions^58^. However, WAS patients may be at increased risk of developing radiation-induced toxicity due to underlying immunodeficiency and potential defects in DNA repair mechanisms^59,60^. Moreover, hematopoietic stem cell transplantation (HSCT) is a potential curative option for WAS patients^61^. Radiation conditioning regimens are commonly administered before HSCT to suppress the recipient’s immune system and enhance the engraftment of donor cells^62^. However, the increased radiation sensitivity of WAS patients raises significant concerns regarding the safety and tolerability of these conditioning regimens. Conducting an assessment of radiation sensitivity in WAS patients before HSCT can assist in risk stratification and treatment planning, thus ensuring the best possible outcomes while minimizing potential complications.

This study demonstrated impaired or reduced DSB repair efficiency and the accumulation of spontaneous and induced DSBs in WAS lymphocytes. However, assessing the fidelity of the DSB repair process itself is another crucial aspect. Errors in the repair process may lead to misrepair products, contributing to chromosomal abnormalities and genomic instability^63^. Cytogenetic markers such as dicentrics and/or chromosomal rearrangements arise from misrepaired DSBs. Investigating these cytogenetic markers can provide further insights into the accuracy of the DSB repair process in WAS patients.

## Conclusions

This study is the first to demonstrate DNA-DSB repair kinetics in WAS patients and their carrier mothers. Our findings reveal variable DSB repair dynamics associated with four different WAS gene mutations, suggesting a potential link between these mutations and altered radiation responses. However, we acknowledge that WAS mutations may not be the sole determinants of cellular radiosensitivity, as the ionizing radiation-induced DNA damage response (DDR) involves a complex network of proteins, and individual variability in other DDR-related genes may also contribute to the observed effects. By analyzing DSB repair in lymphocytes, we provide important insights into repair efficiency and the accumulation of unrepaired DSBs, which may underlie genomic instability in WAS patients. These findings emphasize the need for further research and may have implications for personalized clinical management of patients with WAS.

## Data Availability

All data generated in this study are included in the article. Additional data supporting the findings are available from the corresponding author (Email ID: rajeshc@barc.gov.in), upon reasonable request.
